# 1,2:5,6-Di-*O*-isopropyl­idene-α-d-3-glucofuranosyl (*R*_p_)-2-(diphenyl­phosphino)ferrocene-1-carboxyl­ate

**DOI:** 10.1107/S1600536809038653

**Published:** 2009-09-30

**Authors:** Petr Štěpnička, Martin Lamač, Ivana Císařová

**Affiliations:** a Department of Inorganic Chemistry, Faculty of Science, Charles University in Prague; Hlavova 2030, 12840 Prague 2, Czech Republic

## Abstract

The title compound, [Fe(C_5_H_5_)(C_30_H_32_O_7_P)], which is an inter­mediate in the synthesis of (*R*_p_)-2-(diphenyl­phos­phino)ferrocene-1-carboxylic acid, crystallizes in the common chiral space group *P*2_1_2_1_2_1_. In general, the mol­ecular geometry is very similar to that of the corresponding 2,1′-bis­(diphenyl­phosphino) congener. The ferrocene unit assumes a regular geometry with the proximal bulky substituents efficiently avoiding mutual spatial contacts. In the crystal, the mol­ecules participate in weak intra- and inter­molecular C—H⋯O inter­actions.

## Related literature

The title compound was prepared according to Breit & Breuninger (2005*a* ▶). For a related structure, see: Lamač *et al.* (2009 ▶). For selected references concerning the use of enantio­pure 2-(diphenyl­phosphino)ferrocene-1-carboxylic acids, see: Longmire *et al.* (2000 ▶, 2002 ▶); You *et al.* (2000 ▶, 2001 ▶); Štěpnička (2002 ▶); Breit & Breuninger (2004 ▶, 2005*b* ▶,*c* ▶); Lamač *et al.* (2007 ▶); Bianchini *et al.* (2008 ▶).
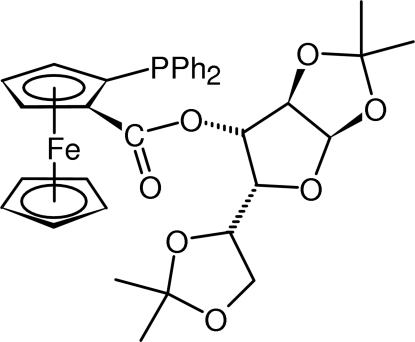


## Experimental

### 

#### Crystal data

[Fe(C_5_H_5_)(C_30_H_32_O_7_P)]
*M* *_r_* = 656.47
Orthorhombic, 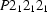

*a* = 10.3488 (1) Å
*b* = 11.5379 (1) Å
*c* = 26.9830 (3) Å
*V* = 3221.86 (6) Å^3^
*Z* = 4
Mo *K*α radiation
μ = 0.57 mm^−1^
*T* = 150 K
0.30 × 0.20 × 0.18 mm

#### Data collection

Nonius KappaCCD diffractometer
Absorption correction: none
68616 measured reflections
7369 independent reflections
6632 reflections with *I* > 2σ(*I*)
*R*_int_ = 0.053

#### Refinement

*R*[*F* ^2^ > 2σ(*F* ^2^)] = 0.042
*wR*(*F* ^2^) = 0.112
*S* = 1.07
7369 reflections
401 parameters
H-atom parameters constrained
Δρ_max_ = 2.00 e Å^−3^
Δρ_min_ = −0.41 e Å^−3^
Absolute structure: Flack (1983 ▶), 3230 Friedel pairs
Flack parameter: −0.012 (16)

### 

Data collection: *COLLECT* (Nonius, 2000 ▶); cell refinement: *SCALEPACK* (Otwinowski & Minor, 1997 ▶); data reduction: *DENZO* (Otwinowski & Minor, 1997 ▶) and *SCALEPACK*; program(s) used to solve structure: *SIR97* (Altomare *et al*., 1999 ▶); program(s) used to refine structure: *SHELXL97* (Sheldrick, 2008 ▶); molecular graphics: *PLATON* (Spek, 2009 ▶); software used to prepare material for publication: *PLATON*.

## Supplementary Material

Crystal structure: contains datablocks I, global. DOI: 10.1107/S1600536809038653/im2141sup1.cif

Structure factors: contains datablocks I. DOI: 10.1107/S1600536809038653/im2141Isup2.hkl

Additional supplementary materials:  crystallographic information; 3D view; checkCIF report

## Figures and Tables

**Table 1:** Hydrogen-bond geometry (Å, °)

*D*—H⋯*A*	*D*—H	H⋯*A*	*D*⋯*A*	*D*—H⋯*A*
C6—H6⋯O34	0.93	2.50	3.357 (4)	153
C9—H9⋯O1^i^	0.93	2.54	3.314 (4)	140

Symmetry code: (i) 
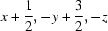
.

**Fig. 1.**
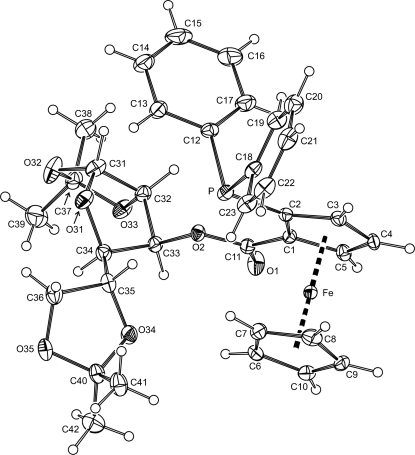
A view of the molecular structure showing the atom numbering scheme and displacement ellipsoids for the non-H atoms at the 30% probability level.

## supplementary crystallographic information

### Comment

1,2:5,6-Di-*O*-isopropylidene-α-D-3-glucofuranosyl 2-(diphenylphosphino)ferrocene-1-carboxylates are useful intermediates in the preparation of optically pure, planar-only chiral 2-(diphenylphosphino)ferrocene-1-carboxylic acids, which already proved to be valuable organometallic synthetic building blocks and chiral auxilliaries (selected references: Longmire *et al.*, 2000 and 2002; You *et al.*, 2000 and 2001; Štěpnička, 2002; Breit & Breuninger, 2004 and 2005*b*,c; Lamač *et al.*, 2007; and Bianchini *et al.*, 2008).

Structural parameters of the title compound (Fig. 1) are very similar to those reported previously for the related bisphosphine derivative, 1,2:5,6-di-*O*-isopropylidene-α-D-3-glucofuranosyl (*R*_p_)-2,1'-bis(diphenylphosphino)ferrocene-1-carboxylate (Lamač *et al.*, 2009). Likwise this reference compound, the ferrocene unit in the title compound is only negligibly tilted (dihedral angle of the mean cyclopentadienyl planes being 2.7 (2)°) and displays similar Fe—ring centroid distances (1.640 (1) and 1.650 (12) Å for the cyclopentadienyl rings C(1–5) and C(6–10), respectively). The ferrocene substituents attached in adjacent positions are directed away from each other so as to avoid spatial contacts and to minimize their steric influence on the ferrocene scaffold (*cf.* the torsion angle C11—C1—C2—P = 0.6 (4)°).

In the solid state, the individual molecules are involved in intra- and intermolecular C—H···O contacts (Table 1).

### Experimental

The title compound was prepared by esterification of racemic 2-(diphenylphosphino)ferrocene-1-carboxylic acid with glucose diacetonide (*i.e.*, 1,2:5,6-di-*O*-isopropylidene-α-D-3-glucofuranose) followed by chromatographic separation of the resulting mixture of diastereoisomers (Breit & Breuninger, 2005*a*). X-ray quality crystals were grown by crystallization from diethyl ether-pentane at -18 °C.

### Refinement

All H-atoms were included in their calculated positions and refined as riding atoms. The unusually high 'positive' residual electron density can be attributed to lone electron pair at phosphorus (*N.B.*: The second largest residual electron density peak has only 0.63 e Å^-3^). Attempted refinement of this largest maximum as a helium atom (two electrons) resulted not only in reasonable geometry (P—*X* = 1.354 (5) Å; C—P—*X* angles = 103.5 (2)–124.6 (2) °) but also in a significant decrease in the *R*-indices (*R* = 0.0314, *wR* = 0.0395% for observed diffractions) and extremes on the residual electron denisty map (0.39, -0.33 e Å^-3^). Besides, the dummy atom is found in a position suitable for the formation of an intramolecular C—H···*X* contact with H35 (*cf.* C35···*X* = 2.14 Å, C35—H35···*X* = 178 °)

### Crystal data

**Table:**

[Fe(C_5_H_5_)(C_30_H_32_O_7_P)]	*F*(000) = 1376
*M**_r_* = 656.47	*D*_x_ = 1.353 Mg m^−^^3^
Orthorhombic, *P*2_1_2_1_2_1_	Mo *K*α radiation, λ = 0.71073 Å
Hall symbol: P 2ac 2ab	Cell parameters from 4167 reflections
*a* = 10.3488 (1) Å	θ = 1.0–27.5°
*b* = 11.5379 (1) Å	µ = 0.56 mm^−^^1^
*c* = 26.9830 (3) Å	*T* = 150 K
*V* = 3221.86 (6) Å^3^	Prism, orange
*Z* = 4	0.30 × 0.20 × 0.18 mm

### Data collection

**Table:**

Nonius KappaCCD diffractometer	6632 reflections with *I* > 2σ(*I*)
Radiation source: fine-focus sealed tube	*R*_int_ = 0.053
horizontally mounted graphite crystal	θ_max_ = 27.5°, θ_min_ = 1.5°
Detector resolution: 9.091 pixels mm^-1^	*h* = −13→13
ω and π scans to fill the Ewald sphere	*k* = −14→14
68616 measured reflections	*l* = −34→34
7369 independent reflections	

### Refinement

**Table:**

Refinement on *F*^2^	Secondary atom site location: difference Fourier map
Least-squares matrix: full	Hydrogen site location: inferred from neighbouring sites
*R*[*F*^2^ > 2σ(*F*^2^)] = 0.042	H-atom parameters constrained
*wR*(*F*^2^) = 0.112	*w* = 1/[σ^2^(*F*_o_^2^) + (0.0602*P*)^2^ + 2.0459*P*] where *P* = (*F*_o_^2^ + 2*F*_c_^2^)/3
*S* = 1.07	(Δ/σ)_max_ = 0.001
7369 reflections	Δρ_max_ = 2.00 e Å^−^^3^
401 parameters	Δρ_min_ = −0.41 e Å^−^^3^
0 restraints	Absolute structure: Flack (1983), 3230 Friedel pairs
Primary atom site location: structure-invariant direct methods	Flack parameter: −0.012 (16)

### Special details

**Table:**

Geometry. All e.s.d.'s (except the e.s.d. in the dihedral angle between two l.s. planes) are estimated using the full covariance matrix. The cell e.s.d.'s are taken into account individually in the estimation of e.s.d.'s in distances, angles and torsion angles; correlations between e.s.d.'s in cell parameters are only used when they are defined by crystal symmetry. An approximate (isotropic) treatment of cell e.s.d.'s is used for estimating e.s.d.'s involving l.s. planes.
Refinement. Refinement of *F*^2^ against all reflections. The weighted *R*-factor *wR* and goodness of fit *S* are based on *F*^2^, conventional *R*-factors *R* are based on *F*, with *F* set to zero for negative *F*^2^. The threshold expression of *F*^2^ > σ(*F*^2^) is used only for calculating *R*-factors(gt) *etc*. and is not relevant to the choice of reflections for refinement.

### Fractional atomic coordinates and isotropic or equivalent isotropic displacement parameters (Å^2^)

**Table:**

	*x*	*y*	*z*	*U*_iso_*/*U*_eq_	
Fe	0.75646 (3)	0.73651 (3)	0.085623 (12)	0.02205 (9)	
P	0.67684 (7)	0.93927 (6)	0.17071 (3)	0.02765 (15)	
O1	0.5336 (2)	0.9165 (2)	0.00514 (7)	0.0406 (5)	
O2	0.48826 (16)	0.95039 (16)	0.08521 (7)	0.0250 (4)	
C1	0.7010 (3)	0.8961 (2)	0.06389 (10)	0.0221 (5)	
C2	0.7578 (3)	0.9010 (2)	0.11293 (9)	0.0224 (5)	
C3	0.8872 (3)	0.8588 (2)	0.10792 (11)	0.0262 (6)	
H3	0.9468	0.8517	0.1336	0.031*	
C4	0.9102 (3)	0.8294 (2)	0.05762 (10)	0.0260 (6)	
H4	0.9869	0.8000	0.0448	0.031*	
C5	0.7963 (3)	0.8527 (2)	0.03036 (11)	0.0264 (6)	
H5	0.7854	0.8416	−0.0035	0.032*	
C6	0.5950 (3)	0.6355 (2)	0.09204 (12)	0.0332 (6)	
H6	0.5102	0.6600	0.0873	0.040*	
C7	0.6644 (3)	0.6387 (2)	0.13739 (11)	0.0353 (7)	
H7	0.6327	0.6648	0.1676	0.042*	
C8	0.7908 (3)	0.5949 (3)	0.12855 (12)	0.0364 (7)	
H8	0.8566	0.5878	0.1518	0.044*	
C9	0.7981 (3)	0.5639 (2)	0.07764 (12)	0.0325 (6)	
H9	0.8698	0.5327	0.0617	0.039*	
C10	0.6769 (3)	0.5888 (2)	0.05542 (12)	0.0337 (6)	
H10	0.6552	0.5764	0.0224	0.040*	
C11	0.5682 (3)	0.9205 (2)	0.04768 (10)	0.0242 (5)	
C12	0.6543 (3)	1.0958 (2)	0.16384 (10)	0.0286 (6)	
C13	0.5657 (3)	1.1494 (3)	0.19509 (13)	0.0374 (7)	
H13	0.5208	1.1057	0.2184	0.045*	
C14	0.5441 (4)	1.2664 (3)	0.19166 (16)	0.0527 (9)	
H14	0.4854	1.3013	0.2131	0.063*	
C15	0.6065 (4)	1.3323 (3)	0.15764 (19)	0.0614 (12)	
H15	0.5906	1.4115	0.1557	0.074*	
C16	0.6950 (4)	1.2801 (3)	0.12553 (17)	0.0568 (10)	
H16	0.7378	1.3245	0.1019	0.068*	
C17	0.7188 (3)	1.1630 (3)	0.12882 (13)	0.0428 (8)	
H17	0.7783	1.1286	0.1076	0.051*	
C18	0.8108 (3)	0.9280 (2)	0.21501 (10)	0.0312 (6)	
C19	0.9024 (3)	1.0140 (3)	0.22259 (11)	0.0352 (7)	
H19	0.9013	1.0804	0.2030	0.042*	
C20	0.9958 (3)	1.0016 (3)	0.25926 (12)	0.0421 (8)	
H20	1.0563	1.0600	0.2643	0.050*	
C21	0.9992 (3)	0.9027 (3)	0.28826 (12)	0.0427 (8)	
H21	1.0623	0.8945	0.3125	0.051*	
C22	0.9091 (4)	0.8164 (3)	0.28114 (12)	0.0429 (8)	
H22	0.9115	0.7498	0.3005	0.051*	
C23	0.8147 (3)	0.8290 (3)	0.24498 (11)	0.0372 (7)	
H23	0.7534	0.7709	0.2406	0.045*	
O31	0.2736 (2)	1.05237 (18)	0.14377 (7)	0.0370 (5)	
O32	0.1519 (3)	1.1833 (2)	0.09835 (9)	0.0525 (7)	
O33	0.2334 (2)	1.11358 (17)	0.02688 (7)	0.0357 (5)	
O34	0.3015 (2)	0.74486 (18)	0.11693 (7)	0.0348 (5)	
O35	0.14038 (19)	0.7207 (2)	0.17217 (8)	0.0374 (5)	
C31	0.2789 (3)	1.1452 (3)	0.11065 (12)	0.0373 (7)	
H31	0.3312	1.2088	0.1240	0.045*	
C32	0.3346 (3)	1.1002 (2)	0.06165 (11)	0.0293 (6)	
H32	0.4140	1.1403	0.0518	0.035*	
C33	0.3552 (2)	0.9699 (2)	0.07100 (10)	0.0260 (6)	
H33	0.3307	0.9231	0.0422	0.031*	
C34	0.2681 (3)	0.9470 (3)	0.11491 (10)	0.0299 (6)	
H34	0.1795	0.9356	0.1030	0.036*	
C35	0.3053 (3)	0.8459 (3)	0.14770 (11)	0.0319 (6)	
H35	0.3920	0.8571	0.1615	0.038*	
C36	0.2076 (3)	0.8218 (3)	0.18907 (12)	0.0401 (7)	
H36A	0.2510	0.8071	0.2203	0.048*	
H36B	0.1487	0.8865	0.1932	0.048*	
C37	0.1424 (3)	1.1947 (3)	0.04633 (12)	0.0348 (7)	
C38	0.1743 (4)	1.3153 (3)	0.02899 (17)	0.0534 (9)	
H38A	0.1140	1.3693	0.0431	0.080*	
H38B	0.1692	1.3185	−0.0065	0.080*	
H38C	0.2603	1.3351	0.0394	0.080*	
C39	0.0093 (3)	1.1574 (4)	0.02956 (19)	0.0577 (10)	
H39A	−0.0124	1.0848	0.0448	0.087*	
H39B	0.0087	1.1486	−0.0058	0.087*	
H39C	−0.0529	1.2150	0.0390	0.087*	
C40	0.2314 (3)	0.6572 (3)	0.14332 (10)	0.0319 (6)	
C41	0.3214 (3)	0.5871 (3)	0.17559 (12)	0.0430 (8)	
H41A	0.3676	0.6381	0.1974	0.065*	
H41B	0.3817	0.5460	0.1551	0.065*	
H41C	0.2722	0.5328	0.1948	0.065*	
C42	0.1585 (4)	0.5848 (3)	0.10663 (13)	0.0467 (8)	
H42A	0.1134	0.5241	0.1237	0.070*	
H42B	0.2180	0.5513	0.0834	0.070*	
H42C	0.0975	0.6325	0.0893	0.070*	

### Atomic displacement parameters (Å^2^)

**Table:**

	*U*^11^	*U*^22^	*U*^33^	*U*^12^	*U*^13^	*U*^23^
Fe	0.02177 (17)	0.02121 (16)	0.02317 (17)	0.00037 (15)	0.00262 (15)	−0.00003 (13)
P	0.0337 (4)	0.0239 (3)	0.0253 (3)	0.0028 (3)	0.0028 (3)	−0.0012 (3)
O1	0.0324 (11)	0.0661 (16)	0.0233 (10)	0.0081 (11)	−0.0023 (8)	−0.0013 (10)
O2	0.0197 (8)	0.0311 (9)	0.0241 (9)	0.0034 (7)	−0.0025 (7)	−0.0028 (8)
C1	0.0224 (12)	0.0207 (11)	0.0232 (12)	0.0016 (9)	0.0020 (10)	−0.0002 (10)
C2	0.0220 (12)	0.0191 (10)	0.0261 (11)	−0.0010 (11)	0.0001 (12)	0.0003 (9)
C3	0.0209 (13)	0.0259 (13)	0.0319 (14)	−0.0008 (10)	−0.0036 (11)	−0.0005 (11)
C4	0.0198 (12)	0.0262 (14)	0.0320 (14)	−0.0001 (10)	0.0077 (11)	0.0037 (11)
C5	0.0253 (13)	0.0272 (13)	0.0267 (13)	−0.0005 (10)	0.0028 (10)	0.0063 (11)
C6	0.0252 (13)	0.0252 (13)	0.0491 (18)	−0.0047 (10)	0.0067 (13)	−0.0001 (13)
C7	0.0468 (18)	0.0262 (14)	0.0330 (15)	−0.0044 (13)	0.0190 (14)	0.0017 (12)
C8	0.0441 (18)	0.0248 (13)	0.0401 (17)	−0.0016 (12)	−0.0016 (13)	0.0099 (12)
C9	0.0349 (14)	0.0191 (12)	0.0436 (17)	0.0031 (10)	0.0117 (12)	−0.0012 (12)
C10	0.0367 (16)	0.0275 (14)	0.0369 (16)	−0.0055 (12)	0.0041 (13)	−0.0066 (12)
C11	0.0246 (13)	0.0237 (13)	0.0243 (13)	0.0003 (10)	0.0006 (10)	0.0012 (10)
C12	0.0291 (14)	0.0256 (12)	0.0310 (14)	0.0000 (11)	−0.0072 (11)	−0.0032 (11)
C13	0.0324 (16)	0.0362 (16)	0.0437 (18)	0.0045 (13)	−0.0024 (13)	−0.0103 (14)
C14	0.0422 (18)	0.0366 (18)	0.079 (3)	0.0080 (16)	−0.0027 (17)	−0.0191 (19)
C15	0.056 (2)	0.0242 (16)	0.104 (4)	0.0048 (16)	−0.023 (2)	−0.0059 (19)
C16	0.068 (2)	0.0303 (17)	0.072 (3)	−0.0042 (17)	0.002 (2)	0.0071 (17)
C17	0.055 (2)	0.0261 (14)	0.0474 (19)	−0.0005 (14)	0.0070 (15)	−0.0008 (13)
C18	0.0401 (15)	0.0304 (14)	0.0231 (13)	0.0070 (12)	0.0024 (11)	−0.0044 (11)
C19	0.0413 (17)	0.0359 (15)	0.0284 (15)	0.0032 (13)	−0.0054 (13)	−0.0015 (12)
C20	0.0406 (17)	0.048 (2)	0.0373 (18)	0.0037 (15)	−0.0029 (15)	−0.0089 (15)
C21	0.0441 (19)	0.055 (2)	0.0285 (15)	0.0132 (16)	−0.0079 (14)	−0.0078 (14)
C22	0.058 (2)	0.0410 (18)	0.0298 (16)	0.0161 (16)	−0.0012 (15)	0.0017 (13)
C23	0.0480 (18)	0.0322 (15)	0.0315 (15)	0.0067 (14)	−0.0010 (14)	−0.0008 (12)
O31	0.0423 (12)	0.0388 (11)	0.0300 (10)	0.0069 (10)	0.0007 (9)	−0.0086 (9)
O32	0.0508 (14)	0.0652 (17)	0.0415 (13)	0.0302 (13)	0.0041 (11)	−0.0036 (11)
O33	0.0382 (12)	0.0353 (10)	0.0337 (10)	0.0132 (10)	−0.0098 (10)	−0.0054 (8)
O34	0.0381 (10)	0.0327 (11)	0.0335 (10)	0.0007 (9)	0.0119 (8)	0.0062 (9)
O35	0.0267 (10)	0.0502 (13)	0.0352 (11)	0.0015 (9)	0.0087 (8)	0.0063 (10)
C31	0.0432 (18)	0.0335 (15)	0.0353 (15)	0.0086 (13)	−0.0087 (13)	−0.0072 (12)
C32	0.0261 (14)	0.0269 (13)	0.0349 (14)	0.0052 (11)	−0.0029 (12)	−0.0012 (11)
C33	0.0212 (12)	0.0279 (13)	0.0289 (13)	0.0031 (10)	−0.0031 (10)	−0.0016 (10)
C34	0.0240 (13)	0.0350 (14)	0.0308 (13)	−0.0001 (12)	−0.0013 (11)	−0.0029 (11)
C35	0.0249 (13)	0.0397 (16)	0.0310 (15)	−0.0010 (12)	0.0031 (11)	0.0030 (12)
C36	0.0383 (17)	0.0512 (19)	0.0307 (15)	−0.0003 (14)	0.0081 (12)	−0.0011 (14)
C37	0.0296 (15)	0.0320 (15)	0.0427 (17)	0.0098 (12)	−0.0025 (13)	−0.0013 (13)
C38	0.048 (2)	0.0327 (16)	0.079 (3)	0.0075 (16)	0.007 (2)	0.0024 (17)
C39	0.0371 (19)	0.050 (2)	0.086 (3)	0.0038 (16)	−0.0101 (19)	0.003 (2)
C40	0.0290 (15)	0.0387 (14)	0.0280 (13)	−0.0009 (12)	0.0043 (12)	0.0079 (11)
C41	0.0363 (16)	0.0530 (19)	0.0398 (17)	0.0037 (15)	0.0064 (14)	0.0161 (15)
C42	0.051 (2)	0.048 (2)	0.0415 (18)	−0.0077 (17)	−0.0051 (16)	0.0030 (15)

### Geometric parameters (Å, °)

**Table:**

Fe—C1	2.016 (3)	C19—C20	1.391 (4)
Fe—C7	2.033 (3)	C19—H19	0.9300
Fe—C8	2.034 (3)	C20—C21	1.383 (5)
Fe—C2	2.035 (2)	C20—H20	0.9300
Fe—C6	2.045 (3)	C21—C22	1.378 (5)
Fe—C3	2.045 (3)	C21—H21	0.9300
Fe—C5	2.047 (3)	C22—C23	1.388 (5)
Fe—C9	2.050 (3)	C22—H22	0.9300
Fe—C10	2.061 (3)	C23—H23	0.9300
Fe—C4	2.062 (3)	O31—C31	1.396 (4)
P—C2	1.824 (3)	O31—C34	1.445 (3)
P—C12	1.831 (3)	O32—C37	1.413 (4)
P—C18	1.835 (3)	O32—C31	1.426 (4)
O1—C11	1.203 (3)	O33—C32	1.415 (3)
O2—C11	1.352 (3)	O33—C37	1.428 (4)
O2—C33	1.447 (3)	O34—C35	1.432 (4)
C1—C5	1.429 (4)	O34—C40	1.434 (3)
C1—C2	1.449 (4)	O35—C40	1.425 (4)
C1—C11	1.469 (4)	O35—C36	1.432 (4)
C2—C3	1.432 (4)	C31—C32	1.533 (4)
C3—C4	1.419 (4)	C31—H31	0.9800
C3—H3	0.9300	C32—C33	1.540 (4)
C4—C5	1.415 (4)	C32—H32	0.9800
C4—H4	0.9300	C33—C34	1.512 (4)
C5—H5	0.9300	C33—H33	0.9800
C6—C10	1.409 (4)	C34—C35	1.514 (4)
C6—C7	1.419 (5)	C34—H34	0.9800
C6—H6	0.9300	C35—C36	1.531 (4)
C7—C8	1.423 (5)	C35—H35	0.9800
C7—H7	0.9300	C36—H36A	0.9700
C8—C9	1.422 (5)	C36—H36B	0.9700
C8—H8	0.9300	C37—C38	1.505 (5)
C9—C10	1.420 (4)	C37—C39	1.512 (5)
C9—H9	0.9300	C38—H38A	0.9600
C10—H10	0.9300	C38—H38B	0.9600
C12—C13	1.391 (4)	C38—H38C	0.9600
C12—C17	1.393 (4)	C39—H39A	0.9600
C13—C14	1.372 (5)	C39—H39B	0.9600
C13—H13	0.9300	C39—H39C	0.9600
C14—C15	1.356 (6)	C40—C42	1.499 (4)
C14—H14	0.9300	C40—C41	1.510 (4)
C15—C16	1.397 (6)	C41—H41A	0.9600
C15—H15	0.9300	C41—H41B	0.9600
C16—C17	1.375 (5)	C41—H41C	0.9600
C16—H16	0.9300	C42—H42A	0.9600
C17—H17	0.9300	C42—H42B	0.9600
C18—C19	1.387 (4)	C42—H42C	0.9600
C18—C23	1.401 (4)		
			
C1—Fe—C7	124.99 (12)	C13—C12—P	117.5 (2)
C1—Fe—C8	161.64 (12)	C17—C12—P	123.8 (2)
C7—Fe—C8	40.94 (13)	C14—C13—C12	120.3 (3)
C1—Fe—C2	41.92 (10)	C14—C13—H13	119.9
C7—Fe—C2	105.79 (11)	C12—C13—H13	119.9
C8—Fe—C2	122.79 (12)	C15—C14—C13	121.3 (4)
C1—Fe—C6	108.19 (11)	C15—C14—H14	119.3
C7—Fe—C6	40.73 (14)	C13—C14—H14	119.3
C8—Fe—C6	68.69 (13)	C14—C15—C16	119.4 (3)
C2—Fe—C6	120.40 (11)	C14—C15—H15	120.3
C1—Fe—C3	69.16 (11)	C16—C15—H15	120.3
C7—Fe—C3	119.41 (12)	C17—C16—C15	120.0 (4)
C8—Fe—C3	105.73 (13)	C17—C16—H16	120.0
C2—Fe—C3	41.07 (11)	C15—C16—H16	120.0
C6—Fe—C3	155.36 (12)	C16—C17—C12	120.3 (3)
C1—Fe—C5	41.19 (10)	C16—C17—H17	119.9
C7—Fe—C5	163.43 (12)	C12—C17—H17	119.8
C8—Fe—C5	154.91 (12)	C19—C18—C23	118.6 (3)
C2—Fe—C5	69.64 (10)	C19—C18—P	124.2 (2)
C6—Fe—C5	126.84 (12)	C23—C18—P	117.1 (2)
C3—Fe—C5	68.24 (11)	C18—C19—C20	120.4 (3)
C1—Fe—C9	156.50 (12)	C18—C19—H19	119.8
C7—Fe—C9	68.36 (12)	C20—C19—H19	119.8
C8—Fe—C9	40.74 (13)	C21—C20—C19	120.4 (3)
C2—Fe—C9	160.49 (12)	C21—C20—H20	119.8
C6—Fe—C9	68.10 (11)	C19—C20—H20	119.8
C3—Fe—C9	124.19 (12)	C22—C21—C20	120.0 (3)
C5—Fe—C9	121.16 (12)	C22—C21—H21	120.0
C1—Fe—C10	121.76 (12)	C20—C21—H21	120.0
C7—Fe—C10	67.98 (12)	C21—C22—C23	119.9 (3)
C8—Fe—C10	68.32 (14)	C21—C22—H22	120.0
C2—Fe—C10	156.46 (12)	C23—C22—H22	120.0
C6—Fe—C10	40.14 (12)	C22—C23—C18	120.7 (3)
C3—Fe—C10	162.01 (12)	C22—C23—H23	119.6
C5—Fe—C10	109.51 (12)	C18—C23—H23	119.6
C9—Fe—C10	40.43 (12)	C31—O31—C34	107.6 (2)
C1—Fe—C4	68.81 (11)	C37—O32—C31	108.9 (2)
C7—Fe—C4	154.44 (13)	C32—O33—C37	108.4 (2)
C8—Fe—C4	119.45 (12)	C35—O34—C40	107.5 (2)
C2—Fe—C4	69.07 (11)	C40—O35—C36	105.8 (2)
C6—Fe—C4	163.34 (12)	O31—C31—O32	110.4 (3)
C3—Fe—C4	40.41 (11)	O31—C31—C32	107.9 (2)
C5—Fe—C4	40.29 (11)	O32—C31—C32	104.5 (2)
C9—Fe—C4	107.72 (11)	O31—C31—H31	111.2
C10—Fe—C4	126.40 (12)	O32—C31—H31	111.2
C2—P—C12	102.19 (12)	C32—C31—H31	111.2
C2—P—C18	101.10 (13)	O33—C32—C31	104.9 (2)
C12—P—C18	103.40 (13)	O33—C32—C33	108.5 (2)
C11—O2—C33	115.1 (2)	C31—C32—C33	103.9 (2)
C5—C1—C2	108.1 (2)	O33—C32—H32	112.9
C5—C1—C11	121.6 (2)	C31—C32—H32	112.9
C2—C1—C11	130.1 (2)	C33—C32—H32	112.9
C5—C1—Fe	70.57 (15)	O2—C33—C34	109.4 (2)
C2—C1—Fe	69.77 (14)	O2—C33—C32	109.1 (2)
C11—C1—Fe	121.91 (19)	C34—C33—C32	102.5 (2)
C3—C2—C1	106.3 (2)	O2—C33—H33	111.8
C3—C2—P	126.4 (2)	C34—C33—H33	111.8
C1—C2—P	127.1 (2)	C32—C33—H33	111.8
C3—C2—Fe	69.83 (14)	O31—C34—C33	104.6 (2)
C1—C2—Fe	68.31 (14)	O31—C34—C35	108.9 (2)
P—C2—Fe	122.15 (13)	C33—C34—C35	116.2 (2)
C4—C3—C2	109.2 (2)	O31—C34—H34	109.0
C4—C3—Fe	70.44 (16)	C33—C34—H34	109.0
C2—C3—Fe	69.10 (14)	C35—C34—H34	109.0
C4—C3—H3	125.4	O34—C35—C34	106.3 (2)
C2—C3—H3	125.4	O34—C35—C36	104.9 (2)
Fe—C3—H3	126.6	C34—C35—C36	113.4 (3)
C5—C4—C3	108.2 (2)	O34—C35—H35	110.6
C5—C4—Fe	69.26 (15)	C34—C35—H35	110.6
C3—C4—Fe	69.15 (16)	C36—C35—H35	110.6
C5—C4—H4	125.9	O35—C36—C35	103.7 (2)
C3—C4—H4	125.9	O35—C36—H36A	111.0
Fe—C4—H4	127.3	C35—C36—H36A	111.0
C4—C5—C1	108.2 (2)	O35—C36—H36B	111.0
C4—C5—Fe	70.45 (15)	C35—C36—H36B	111.0
C1—C5—Fe	68.24 (15)	H36A—C36—H36B	109.0
C4—C5—H5	125.9	O32—C37—O33	105.0 (2)
C1—C5—H5	125.9	O32—C37—C38	112.3 (3)
Fe—C5—H5	127.0	O33—C37—C38	110.3 (3)
C10—C6—C7	108.1 (3)	O32—C37—C39	109.5 (3)
C10—C6—Fe	70.55 (17)	O33—C37—C39	107.7 (3)
C7—C6—Fe	69.20 (16)	C38—C37—C39	111.8 (3)
C10—C6—H6	126.0	C37—C38—H38A	109.5
C7—C6—H6	126.0	C37—C38—H38B	109.5
Fe—C6—H6	125.9	H38A—C38—H38B	109.5
C6—C7—C8	108.2 (3)	C37—C38—H38C	109.5
C6—C7—Fe	70.07 (16)	H38A—C38—H38C	109.5
C8—C7—Fe	69.57 (17)	H38B—C38—H38C	109.5
C6—C7—H7	125.9	C37—C39—H39A	109.5
C8—C7—H7	125.9	C37—C39—H39B	109.5
Fe—C7—H7	126.0	H39A—C39—H39B	109.5
C9—C8—C7	107.5 (3)	C37—C39—H39C	109.5
C9—C8—Fe	70.20 (17)	H39A—C39—H39C	109.5
C7—C8—Fe	69.49 (17)	H39B—C39—H39C	109.5
C9—C8—H8	126.2	O35—C40—O34	104.1 (2)
C7—C8—H8	126.2	O35—C40—C42	108.4 (3)
Fe—C8—H8	125.6	O34—C40—C42	108.6 (2)
C10—C9—C8	108.0 (3)	O35—C40—C41	111.6 (2)
C10—C9—Fe	70.23 (16)	O34—C40—C41	110.6 (2)
C8—C9—Fe	69.06 (16)	C42—C40—C41	113.1 (3)
C10—C9—H9	126.0	C40—C41—H41A	109.5
C8—C9—H9	126.0	C40—C41—H41B	109.5
Fe—C9—H9	126.3	H41A—C41—H41B	109.5
C6—C10—C9	108.2 (3)	C40—C41—H41C	109.5
C6—C10—Fe	69.31 (16)	H41A—C41—H41C	109.5
C9—C10—Fe	69.35 (17)	H41B—C41—H41C	109.5
C6—C10—H10	125.9	C40—C42—H42A	109.5
C9—C10—H10	125.9	C40—C42—H42B	109.5
Fe—C10—H10	127.0	H42A—C42—H42B	109.5
O1—C11—O2	122.8 (2)	C40—C42—H42C	109.5
O1—C11—C1	123.7 (3)	H42A—C42—H42C	109.5
O2—C11—C1	113.5 (2)	H42B—C42—H42C	109.5
C13—C12—C17	118.7 (3)		
			
C5—C1—C2—C3	−0.6 (3)	C21—C22—C23—C18	0.8 (5)
C11—C1—C2—C3	175.0 (3)	C19—C18—C23—C22	−0.7 (4)
C5—C1—C2—P	−175.07 (19)	P—C18—C23—C22	−176.8 (2)
C11—C1—C2—P	0.6 (4)	C34—O31—C31—O32	89.5 (3)
C12—P—C2—C3	114.0 (2)	C34—O31—C31—C32	−24.1 (3)
C18—P—C2—C3	7.5 (3)	C37—O32—C31—O31	−131.8 (3)
C12—P—C2—C1	−72.6 (2)	C37—O32—C31—C32	−16.0 (3)
C18—P—C2—C1	−179.1 (2)	C37—O33—C32—C31	18.8 (3)
C1—C2—C3—C4	0.3 (3)	C37—O33—C32—C33	129.4 (2)
P—C2—C3—C4	174.88 (19)	O31—C31—C32—O33	115.8 (3)
C2—C3—C4—C5	0.0 (3)	O32—C31—C32—O33	−1.8 (3)
C3—C4—C5—C1	−0.4 (3)	O31—C31—C32—C33	1.9 (3)
C2—C1—C5—C4	0.6 (3)	O32—C31—C32—C33	−115.7 (3)
C11—C1—C5—C4	−175.5 (2)	C11—O2—C33—C34	153.8 (2)
C10—C6—C7—C8	0.7 (3)	C11—O2—C33—C32	−94.9 (3)
C6—C7—C8—C9	−0.5 (3)	O33—C32—C33—O2	152.3 (2)
C7—C8—C9—C10	0.1 (3)	C31—C32—C33—O2	−96.4 (3)
C7—C6—C10—C9	−0.7 (3)	O33—C32—C33—C34	−91.8 (3)
C8—C9—C10—C6	0.4 (3)	C31—C32—C33—C34	19.5 (3)
C33—O2—C11—O1	3.9 (4)	C31—O31—C34—C33	37.1 (3)
C33—O2—C11—C1	−177.2 (2)	C31—O31—C34—C35	161.9 (2)
C5—C1—C11—O1	−7.2 (4)	O2—C33—C34—O31	81.6 (3)
C2—C1—C11—O1	177.7 (3)	C32—C33—C34—O31	−34.1 (3)
C5—C1—C11—O2	174.0 (2)	O2—C33—C34—C35	−38.4 (3)
C2—C1—C11—O2	−1.1 (4)	C32—C33—C34—C35	−154.1 (2)
C2—P—C12—C13	163.4 (2)	C40—O34—C35—C34	−132.7 (2)
C18—P—C12—C13	−91.8 (2)	C40—O34—C35—C36	−12.2 (3)
C2—P—C12—C17	−15.4 (3)	O31—C34—C35—O34	−178.5 (2)
C18—P—C12—C17	89.3 (3)	C33—C34—C35—O34	−60.8 (3)
C17—C12—C13—C14	−0.8 (5)	O31—C34—C35—C36	66.7 (3)
P—C12—C13—C14	−179.8 (3)	C33—C34—C35—C36	−175.6 (3)
C12—C13—C14—C15	0.9 (5)	C40—O35—C36—C35	30.5 (3)
C13—C14—C15—C16	−0.2 (6)	O34—C35—C36—O35	−11.1 (3)
C14—C15—C16—C17	−0.5 (6)	C34—C35—C36—O35	104.6 (3)
C15—C16—C17—C12	0.5 (6)	C31—O32—C37—O33	27.8 (3)
C13—C12—C17—C16	0.2 (5)	C31—O32—C37—C38	−92.1 (3)
P—C12—C17—C16	179.0 (3)	C31—O32—C37—C39	143.1 (3)
C2—P—C18—C19	81.3 (3)	C32—O33—C37—O32	−29.0 (3)
C12—P—C18—C19	−24.2 (3)	C32—O33—C37—C38	92.2 (3)
C2—P—C18—C23	−102.9 (2)	C32—O33—C37—C39	−145.6 (3)
C12—P—C18—C23	151.6 (2)	C36—O35—C40—O34	−38.8 (3)
C23—C18—C19—C20	0.0 (4)	C36—O35—C40—C42	−154.2 (3)
P—C18—C19—C20	175.7 (2)	C36—O35—C40—C41	80.5 (3)
C18—C19—C20—C21	0.7 (5)	C35—O34—C40—O35	31.3 (3)
C19—C20—C21—C22	−0.5 (5)	C35—O34—C40—C42	146.6 (3)
C20—C21—C22—C23	−0.2 (5)	C35—O34—C40—C41	−88.6 (3)

### Hydrogen-bond geometry (Å, °)

**Table:**

*D*—H···*A*	*D*—H	H···*A*	*D*···*A*	*D*—H···*A*
C6—H6···O34	0.93	2.50	3.357 (4)	153
C9—H9···O1^i^	0.93	2.54	3.314 (4)	140

Symmetry codes: (i) *x*+1/2, −*y*+3/2, −*z*.
